# Accumulating Evidence for Axonal Translation in Neuronal Homeostasis

**DOI:** 10.3389/fnins.2017.00312

**Published:** 2017-05-31

**Authors:** Emily L. Spaulding, Robert W. Burgess

**Affiliations:** ^1^The Jackson LaboratoryBar Harbor, ME, United States; ^2^Graduate School of Biomedical Sciences and Engineering, University of MaineOrono, ME, United States

**Keywords:** axon degeneration, local translation, peripheral neuropathy, axonal transport, hereditary sensory and motor neuropathy

## Abstract

The specialized structure of the neuron requires that homeostasis is sustained over the meter or more that may separate a cell body from its axonal terminus. Given this impressive distance and an axonal volume that is many times that of the cell body, how is such a compartment grown during development, re-grown after injury, and maintained throughout adulthood? While early answers to these questions focused on the local environment or the cell soma as supplying the needs of the axon, it is now well-established that the axon has some unique needs that can only be met from within. Decades of research have revealed local translation as an indispensable mechanism of axonal homeostasis during development and regeneration in both invertebrates and vertebrates. In contrast, the extent to which the adult, mammalian axonal proteome is maintained through local translation remains unclear and controversial. This mini-review aims to highlight important experiments that have helped to shape the field of axonal translation, to discuss conceptual arguments and recent evidence that supports local translation as important to the maintenance of adult axons, and to suggest experimental approaches that have the potential to further illuminate the role of axonal translation in neuronal homeostasis.

## Somal provision of the axonal proteome

In the early twentieth century popular opinion stated that the axon drew the majority of its nutrients from the local environment. Classic experiments by Weiss and Hiscoe challenged this idea by providing observable evidence for the directed movement of material from the neuronal soma to the axon terminal (Weiss and Hiscoe, 1948). Rat peripheral nerves were crushed, ligated, and allowed to regenerate. Within days, the axon segment just proximal to the ligation became swollen and enlarged. Upon removal of the ligation, the accumulated axoplasm moved from the soma to the axon, suggesting the presence of a dynamic communication system between the cell body and the axon and establishing the field of axonal transport.

Soon the idea that the neuronal soma supplied the axonal proteome prevailed. Radiolabeled amino acids systemically injected into rats were incorporated into new proteins in the soma and at the base of large dendrites within minutes of injection, but were observed in the axon hillock 1 day after injection, in the ventral root 2 days after injection, and 20 mm down the sciatic nerve 16 days after injection (Droz and Leblond, 1963). It was concluded that proteins are continuously synthesized in the cell body and at the base of large dendritic spines, and are subsequently transported into distal dendrites and axons. Although background from supporting cells would have made it difficult to observe low signals in axons, this evidence supported the idea that would dominate the field for the next several decades; translation does not occur in axons.

## Dendritic translation reveals the potential for local control of the proteome in neurons

The earliest electron microscopy studies of neurons established the soma as the primary site of protein synthesis, based on the presence of well-defined endoplasmic reticulum (ER) and a large number of polyribosomes (Palay and Palade, 1955). Thus, the observation of newly synthesized proteins at the base of large dendrites led to the search and eventual identification of additional components of translation machinery in this compartment. The presence of polyribosomes and specific mRNA species, including microtubule associated protein 2 (MAP2) and the alpha subunit of calcium/calmodulin-dependent protein kinase II (CAMKIIα) were soon identified (Steward and Levy, 1982; Steward, 1983; Caceres et al., 1988).

Decades after the first behavioral studies in mammals showed that memory is dependent upon new protein synthesis, Kang and Schuman assigned physiological significance to dendritic translation by showing it is a requirement for synaptic plasticity in the mammalian hippocampus (Flexner et al., 1963; Kang and Schuman, 1996). Aakalu et al. provided visual evidence of dendritic translation in mammalian neurons using a reporter in which the coding sequence of green fluorescence protein (GFP) was flanked by the 5′ and 3′ untranslated ends of CAMKIIα, resulting in dendritic mRNA localization (Aakalu et al., 2001). Stimulation of cultured rat hippocampal neurons with Brain-derived neurotrophic factor (BDNF) resulted in dendritic synthesis of the GFP reporter, which was abolished by treatment with protein synthesis inhibitors. Possible diffusion from the cell body was eliminated by transection of dendrites from the soma and by including a membrane tether to the reporter protein. This study supplied the evidence needed to acknowledge dendritic translation as a mechanism of neuronal homeostasis.

## Axons possess translation machinery

While the field of dendritic translation advanced, the search for evidence of axonal translation despite the apparent absence of polyribosomes and rRNA in the axon continued (Lasek et al., 1973). A series of *in vitro* metabolic labeling studies in goldfish, squid, and rabbit established that proteins can be synthesized in invertebrate and vertebrate axons (Koenig, 1967; Giuditta et al., 1968; Edstrom and Sjostrand, 1969). Eventually, more sensitive techniques enabled the identification of rRNA, mRNA, and actively translating polysomes in squid giant axons (Giuditta et al., 1980, 1986, 1991). In mammalian axons, ribosomes were identified at embryonic stages both in culture and *in vivo* (Tennyson, 1970; Bunge, 1973; Bassell et al., 1998). Polyribosomes were observed by electron microscopy in the axonal initial segment of mature mammalian central nervous system (CNS) neurons, although not in myelinated sections. Polyribosomes were tightly associated with synapses, suggesting that axonal translation may occur during times of extensive synaptic growth, such as development (Steward and Ribak, 1986).

Electron microscopy failed to detect rough ER or golgi apparatus in vertebrate axons, raising the question of whether or not axons have the ability to process or secrete locally synthesized proteins (Tennyson, 1970; Bunge, 1973). However, the Twiss lab addressed this question using cultured rat sensory neurons. Their extensive studies have shown that (1) ER and golgi components needed for classical protein synthesis and secretion are present in the axon, (2) ER chaperone proteins can be axonally translated, and (3) axons can target locally synthesized proteins to the membrane (Willis et al., 2005; Merianda et al., 2009). This evidence strongly suggests that neurons can post-translationally modify and secrete axonally synthesized proteins, although the associated machinery may exist in very small quantities or adopt unique morphologies in the axon, perhaps explaining how the axon is able to maintain the energetic burden of translation machinery.

## Axonal translation occurs during nervous system development

Given that axonal ribosomes are present in embryonic axons and preferentially associated with synapses, many of the earliest studies in the field of axonal translation were related to its role in neuronal development. Axonal mRNA localization was revealed as a general mechanism of protein sorting and proteome management in the developing axon (Jung et al., 2012). In growing neurons, mRNA is sorted to neuronal processes in granules that also contain ribosomal subunits and translation factors (Knowles et al., 1996; Olink-Coux and Hollenbeck, 1996). β-actin was among the first such mRNA species to be identified as enriched within growth cones and axonal processes of developing neurons, and the axonal synthesis of actin protein in embryonic neurons was established soon after (Bassell et al., 1998; Eng et al., 1999).

It is now known that axonal translation is important for many aspects of neuronal development. Some neurotrophins can induce growth cones to turn toward their source in an actin-dependent manner (Zheng et al., 1996; Ming et al., 1997). The finding that β-actin mRNA is translated in the axon made this mechanism a good candidate for how axons can quickly and independently modulate the cytoskeleton for growth cone turning during development. Confirming this hypothesis, stimulation of either embryonic or adult neurons with neutrotrophins increases the transport of β-actin mRNA into the axon (Zhang et al., 1999, 2001; Willis et al., 2005). A directional gradient of netrin-1 induces translation of β-actin that directly precedes attractive growth cone turning (Leung et al., 2006). Repulsion is just as important as attraction for axonal pathfinding during development, and axonal translation contributes to this phenomenon as well. The guidance cue Semaphorin 3A results in axonal translation of RhoA mRNA and subsequent collapse of the growth cone, preventing the axon from innervating incorrect targets (Wu et al., 2005). Overall neuronal growth and size-sensing is also dependent upon mRNA localization and axonal translation. In growing sensory neurons importin β1 mRNA is anterogradely transported to the axon, where it associates with ribosomes. Perturbation of this localization by 3′ UTR knockout or by sequestration of the importin β1-ribonucleoprotein complex to the cell body results in significantly longer axons (Perry et al., 2016). Finally, axonal protein synthesis is essential for localized BDNF-induced synaptic potentiation in developing neurons (Zhang and Poo, 2002). Thus, axonal translation is a mechanism by which growing neurons correctly pathfind, innervate target tissues, sense their own size, and modulate synaptic strength during development.

## Axonal translation occurs during nervous system regeneration

Immunohistological studies eventually revealed that mature mammalian axons of the peripheral nervous system contain ribosomal proteins and rRNA, which are irregularly distributed and located close to the plasma membrane, possibly explaining the difficulty in identifying them (Koenig et al., 2000). The importance of axonal translation to neuronal growth during development and the identification of ribosomes in adult peripheral axons raised the possibility that axonal translation plays an additional role during peripheral nerve regeneration. Adult rat dorsal root ganglion (DRG) cells were shown to regenerate neuronal processes *in vitro* after *in vivo* axonal crush by regulating the translation of an existing pool of mRNAs (Twiss et al., 2000). Moreover, translation of these mRNAs within the axon itself is required for normal regeneration *in vitro*. *In vivo*, rat motor axons of the sciatic nerve isolated 7 days after a crush injury contain translation factors, ribosomal proteins, and rRNA (Zheng et al., 2001). Whether translation components originate from the neuron or from glial cells is still under question. Glia-to-axon transfer of proteins occurs in squid giant axons transected from their cell bodies (Lasek et al., 1977). In perfused squid axons cell-to-cell transfer of RNA occurs upon stimulation of glial receptors by axonal neurotransmitters, indicating that signaling from active or injured axons can induce glial cells to provide axonal translation components (Eyman et al., 2007). In mammals, the *in vivo* transfer of ribosomal components from Schwann cells to peripheral axons after injury suggests the presence of a dynamic collaboration between these cell types during regeneration (Court et al., 2008, 2011). Independent of the origin of translation machinery in uninjured axons, the glia-axon collaboration during injury conditions, along with increased aggregation of axonal ER components, suggests an increased capacity for axonal translation, processing, and secretion of newly synthesized proteins during regeneration (Merianda et al., 2009; Court et al., 2011).

## Conceptual arguments for axonal translation in neuronal homeostasis

**Figure 1:**
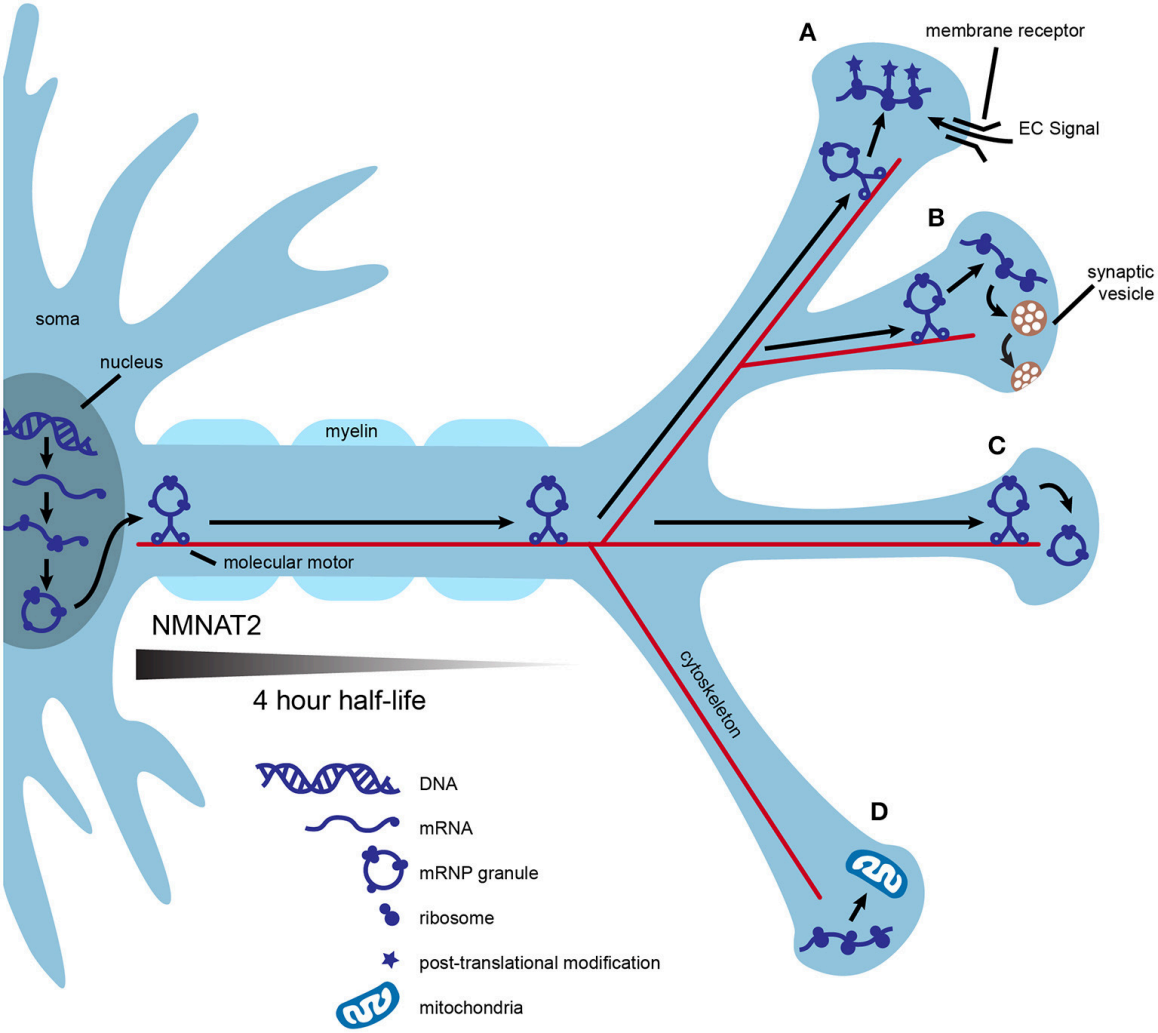
Local translation may contribute to axonal efficiency and compartmentalization. mRNA is transcribed in the nucleus, packaged into mRNP granules, and transported by multiple molecular motors down the axon. Granules are transported into some synapses and mRNA is immediately translated into polypeptides **(A,B)**. Nascent polypeptides may present the opportunity for unique post-translational modification, as in synapse **(A)** or may be used to fulfill specific activity-based needs of individual synapses, as in **(B)**. Local translation of some mRNAs may be controlled at the synaptic level by the interaction of local extracellular signals, such as neurotrophins, with membrane receptors **(A)**. Alternatively, mRNP granules can be transported into synapses and stored for later use **(C)**, or excluded from other synapses **(D)**. Local translation of mitochondrial proteins could help to maintain a healthy supply of axonal mitochondria **(D)**. Axonal survival factors may also be translated locally. For example, NMNAT2 has a half-life of 4 h, and even if transported via fast axonal transport, would not make it to the most distance synapses before significant degradation.

One of the most common conceptual arguments for the role of axonal translation in neuronal maintenance centers around efficiency. Mature mammalian neurons are the largest cells in the body and are highly compartmentalized. Transport of mRNA to distant locations within a neuron followed by local translation may be more efficient than transportation and storage of proteins. One mRNA molecule can be translated many times, allowing for communication with the use of fewer resources, fewer space restrictions, and less risk of aberrant protein accumulation. In addition, nascent proteins may provide opportunities for unique post-translational processing crucial to specific functions in the mature axonal compartment (Figures 1A,C; Jung et al., 2012; Perry and Fainzilber, 2014).

Axonal translation could also confer molecular flexibility at individual synapses. This could be especially useful during periods of high synaptic activity, providing synapse-specific, “on demand” adaptations to the proteome. Replenishment of proteins used for neurotransmission may be needed at some synapses after intense use such as during motor activity or learning. An example of this is found in growing axons, in which the membrane-bound receptor DCC physically associates with translation machinery and mediates local protein synthesis upon stimulation with netrin (Tcherkezian et al., 2010). In this example, the availability of extracellular signals coupled with the expression of presynaptic membrane receptors provides translational control at the level of individual synapses (Figure 1B).

Local translation may help axons maintain a healthy supply of functional mitochondria. Mutations that affect either the function or transport of axonal mitochondria result in neurodegeneration (Schwarz, 2013; Pease and Segal, 2014). Nigrostriatal dopamine neurons have extensive axonal arbors, estimated to form up to 245,000 synapses (Matsuda et al., 2009; Bolam and Pissadaki, 2012). Given that mitochondria are enriched at synapses, the cell body may be unable to synthesize the complete nuclear mitochondrial proteome at a rate sufficient for an uninterrupted supply of axonal mitochondria (Court and Coleman, 2012; Schwarz, 2013). Proteins of the inner and outer mitochondrial membranes possess different turnover rates, thus, axonally translated mitochondrial proteins may also allow for finer control of mitochondrial replenishment (Beattie, 1969). In support of axonal synthesis of nuclear-encoded mitochondrial proteins, rat superior cervical ganglia (SCG) axons contain mRNA for several of these proteins and inhibition of axonal protein synthesis decreases mitochondrial membrane potential (Hillefors et al., 2007). SCG axons also contain the micro RNA (miR), miR-338, which is known to post-transcriptionally modulate the expression of cytochrome c oxidase IV (COXIV), a nuclear protein important to oxidative phosphorylation. Overexpression of miR-338 in the axon reduces COXIV protein levels, mitochondrial oxygen consumption, and axonal ATP levels (Figure 1D; Aschrafi et al., 2008).

Finally, axonal translation may provide a local supply of essential axon survival factors. Neurotrophins are important regulators of axonal survival that rely on both transported and locally translated proteins to exert their protective effects. For example, stimulation of axons by neurotrophins coordinates transcription of the antiapoptotic gene *bcl-w* with transport of bcl-w mRNA to the axon and subsequent local translation (Cosker et al., 2013). Because the inhibition of axonal protein synthesis with cycloheximide abolishes the protective effects of neurotrophins, local synthesis of other axonal survival proteins is likely (Pazyra-Murphy et al., 2009; Pease and Segal, 2014). A possible example is nicotinamide nucleotide adenylyltransferase 2 (NMNAT2), an essential axon survival factor with a half-life of only 4 h (Gilley and Coleman, 2010). Even if NMNAT2 were transported by fast axonal transport, estimated to move cargo at a speed of 400 mm/day, the protein would only travel ~67 mm before 50 percent degradation (Hirokawa et al., 2010). This would result in vanishingly low levels of NMNAT2 in the most distal axons in large mammals, including humans. Distinct neuronal types may require different balances of transported vs. locally translated NMNAT2 (Figure 1).

## Recent evidence for local translation in adult mammalian axons

Despite these arguments, the physiological significance of axonal translation in nervous system maintenance remains ambiguous. Asymmetrical mRNA localization is a commonly utilized communication strategy for many types of mature polarized cells. (Xing and Bassell, 2013). It is tempting to hypothesize that the longest axons *in vivo*, such as mature sensory and motor peripheral axons, may be the most reliant on mRNA transport and local translation for homeostasis. Mature sensory axons possess a complex repertoire of mRNA, and it is suspected that the microtubule stabilizing agent, Paclitaxel, causes sensory neuropathy at least in part by impairing axonal transport (Scripture et al., 2006; Gumy et al., 2011). More direct evidence of neurodegeneration as a result of dysregulation of mRNA transport is found with mutations in the RNA binding protein, SMN1, which cause the severe motor neuron disease, spinal muscular atrophy (Wang et al., 2016). Mutations in at least five members of the ubiquitously expressed family of proteins, tRNA synthetases, cause the specific degeneration of sensory and motor axons, supporting the idea that local translation of transported mRNA is crucial for axonal maintenance (Antonellis and Green, 2008).

A challenge in establishing axonal translation in mature mammalian neurons is studying axons in isolation from their cell bodies and other supporting cell types *in vivo*. The genetic method, translating ribosome affinity purification (TRAP), now allows for axonally-derived populations of ribosomes and their associated RNA to be analyzed without fear of contamination from other cell types. Shigeoka, et al. used the RiboTag knockin mouse line, in which Cre-mediated recombination results in expression of a triple HA-tagged ribosomal protein, RPL22 (Sanz et al., 2009; Shigeoka et al., 2016). Ribosome-bound mRNAs in retinal ganglion cell (RGC) axons were isolated from their CNS targets in developing and adult mice. Comparison of cell somas in the retina to axons in the brain revealed distinct populations of ribosome-associated mRNAs in axons. Axons at all ages were enriched for the gene ontology (GO) terms “cellular metabolism” and “mitochondrial respiratory chain,” suggesting that mitochondrial proteins are indeed locally translated in developing and adult axons. Analysis of developmental stages revealed that axonal translation is intimately associated with RGC circuit assembly. In contrast, adult axons are enriched for transcripts related to the maintenance of neurotransmission, including components of the trans-SNARE complex, glutamate receptors, and neurotrophin receptors. NMNAT2 transcript is associated with ribosomes in both developing and adult axons, but more highly enriched at the adult stage. Neurotrophin-induced survival signals, including components of the CREB and STAT3 pathways, are also enriched in the adult axon. This study provides key evidence that axonal translation in adult RGCs supports metabolic function, neurotransmission, axon survival, and many other aspects of axonal homeostasis.

## Approaches to illuminate the role of axonal translation in neuronal homeostasis

Additional studies are needed to provide a complete picture of the physiological relevance of translation in adult mammalian axons *in vivo*, and to definitively show when and where axonal translation is occurring. Visualization of nascent protein would provide the strongest evidence for axonal translation. Similar to the approach used by Aakalu et al. (2001) for the visualization of dendritic translation, Willis et al. used the 3′ UTR of rat β-actin mRNA to drive axonal localization of a GFP reporter protein containing a membrane tether. GFP protein was indeed locally synthesized in cultured axons of adult, rat DRGs and mRNA was transported to axons of adult central and peripheral neurons *in vivo* (Willis et al., 2007, 2011). Single molecule translation imaging (SMTI) was recently used to determine the temporal and spatial dynamics of β-actin translation in cultured *Xenopus* RGC axons (Strohl et al., 2017). The fast-folding, fast-bleaching fluorescent protein, Venus, was fused to the full-length β-actin sequence, and translation was visualized under baseline conditions and with Netrin-1 stimulation. Real-time visualization of axonally translated protein *in vivo* in a mammalian system is the next step. *In vivo* expression of a construct similar to that used by Strohl et al. could be driven in neurons and temporally and spatially analyzed using SMTI. TRAP studies could provide a list of axonally translated proteins in a given cell type with which to drive axonal localization of the reporter.

While the above experiment would unequivocally demonstrate axonal translation *in vivo*, it is limited in scope to demonstrating the synthesis of one protein at a time. New *in vivo* techniques for large-scale labeling of nascent proteins will be invaluable to the full characterization of the axonal proteome. Ultimately, a complete catalog of axonally translated proteins at developmental and adult stages of life in each neuronal cell type will be needed to understand the intricacies of axonal translation, its contribution to neuronal homeostasis, and how its disruption can lead to neurodegeneration.

## Author contributions

RB assisted in the planning and editing of the paper, ES researched and wrote the manuscript.

### Conflict of interest statement

The authors declare that the research was conducted in the absence of any commercial or financial relationships that could be construed as a potential conflict of interest.
